# Supplementary material for: A diminutive new basilosaurid whale reveals the trajectory of the cetacean life histories during the Eocene
Source: Commun Biol. 2023 Aug 10;6:707. doi: 10.1038/s42003-023-04986-w (PMC10415296; doi:10.1038/s42003-023-04986-w)
Supplement: Supplementary file 6 — Supplementary Data 3 [file 42003_2023_4986_MOESM6_ESM.zip › Supplementary Data 3/Supplementary Data 1_BTD_ASR/trait_0154_tree.plot.pdf]

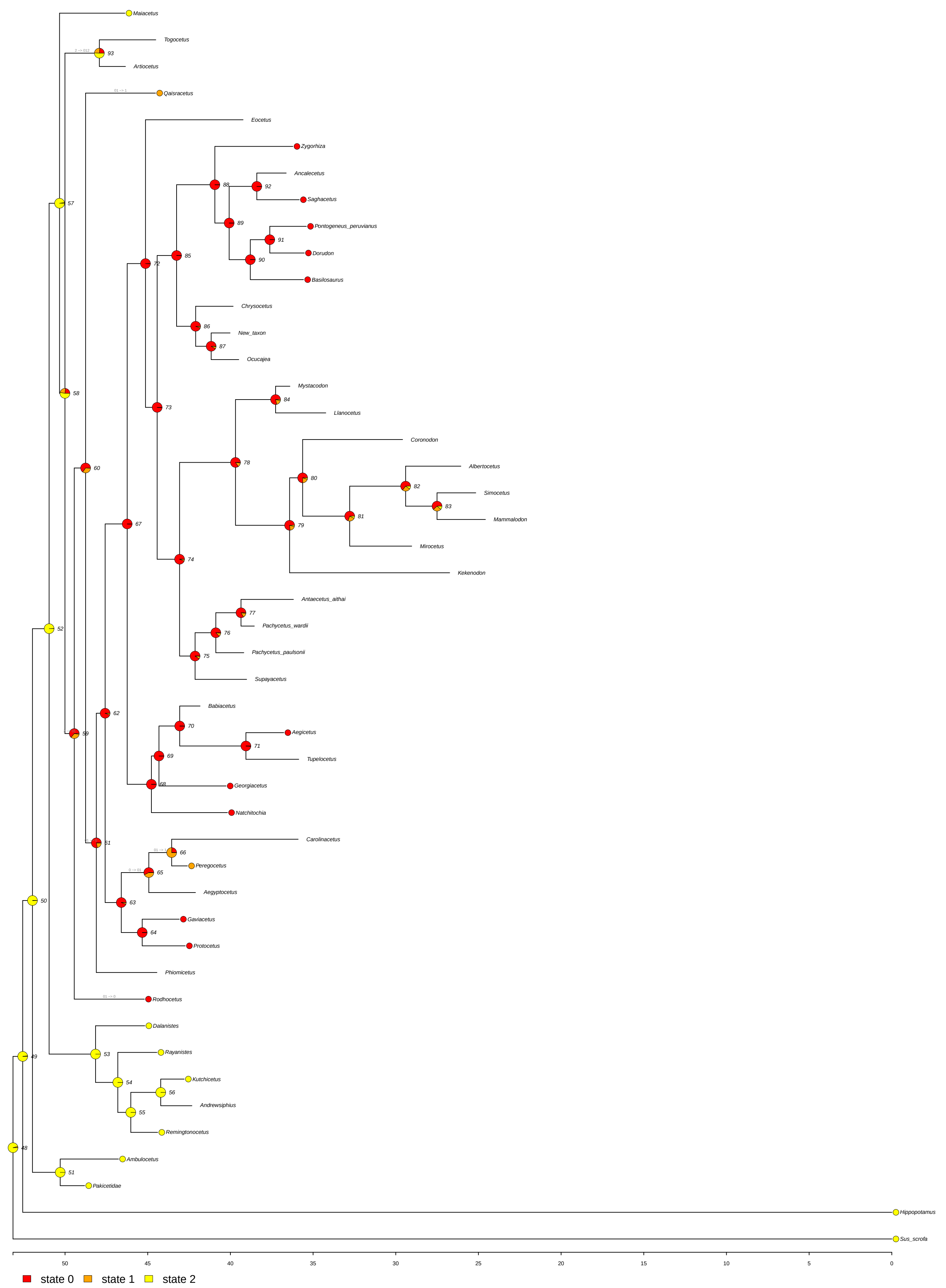

Maiacetus
Togocetus
2 -> 012
93
Artiocetus
01 -> 1
Qaisracetus
Eocetus
Zygorhiza
Ancalecetus
88
92
Saghacetus
57
Pontogeneus_peruvianus
89
91
Dorudon
90
72
85
Basilosaurus
Chrysocetus
86
New_taxon
87
Ocucajea
Mystacodon
58
84
Llanocetus
73
Coronodon
60
78
Albertocetus
80
82
Simocetus
83
81
Mammalodon
67
79
Mirocetus
74
Kekenodon
Antaecetus_aithai
77
52
76
Pachycetus_wardii
Pachycetus_paulsonii
75
Supayacetus
Babiacetus
62
59
70
Aegicetus
71
Tupelocetus
69
68
Georgiacetus
Natchitochia
Carolinacetus
61
01 -> 1
66
Peregocetus
0 -> 01
65
Aegyptocetus
50
63
Gaviacetus
64
Protocetus
Phiomicetus
01 -> 0
Rodhocetus
Dalanistes
53
49
Rayanistes
54
Kutchicetus
56
55
Andrewsiphius
Remingtonocetus
48
Ambulocetus
51
Pakicetidae
Hippopotamus
Sus_scrofa
50
45
40
35
30
25
20
15
10
5
0
state 0
state 1
state 2
